# GADD45G as a novel prognostic biomarker and therapeutic target in glioma: integrative analysis of bulk and single-cell RNA sequencing

**DOI:** 10.3389/fonc.2025.1608710

**Published:** 2025-09-11

**Authors:** Cong Shen, Haiping Cai, Chengliang Mao, Jiahao Yang, Kai Tang

**Affiliations:** ^1^ Shantou University Medical College, Shantou, China; ^2^ Department of Neurosurgery, Guangdong Provincial People’s Hospital (Guangdong Academy of Medical Sciences), Southern Medical University, Guangzhou, China

**Keywords:** GADD45G, gliomas, prognostic biomarker, therapeutic target, EMT-like

## Abstract

**Background:**

Gliomas make up almost half of primary central nervous system tumors. Despite advancements in surgery and neuro-oncology, developing an effective treatment remains challenging. The protein Growth Arrest and DNA-Damage-Inducible, Gamma (GADD45G) is crucial for key cellular functions like DNA repair, genomic stability, and apoptosis. While GADD45G dysregulation has been found in various cancers, its role in glioma is still unclear.

**Methods:**

We analyzed unified pan-cancer datasets (TCGA, TARGET, GTEx) from UCSC Xena and integrated glioma data from CGGA and GEO (GSE108476). Prognostic value was assessed via multivariate Cox regression and Kaplan-Meier survival analysis using Gliovis. Single-cell RNA-seq data (GSE103224, GSE138794, GSE173278) were processed with Seurat (R 4.2.2), with Harmony for batch correction and UMAP for visualization. Malignant subclusters were annotated using marker genes. Functional enrichment and cell-type proportion estimation were conducted. Single-cell analysis revealed GADD45G expression patterns and identified its top correlated genes in malignant glioblastoma cells. Overexpression of GADD45G was performed to investigate its impact on cell function. Western blot analysis was used to examine the role of GADD45G in glioma cell invasion and migration.

**Results:**

Through comprehensive analysis across multiple datasets, it was found that GADD45G expression is higher in glioma patients compared to normal individuals, and its expression is generally higher in lower-grade gliomas than in glioblastoma. Cox regression analysis indicated that GADD45G has a protective effect. Survival curves further demonstrated that elevated GADD45G levels are associated with improved overall survival in patients. In this study, we identified four highly heterogeneous GBM cell subpopulations using single-cell data. The MES-like cells was significantly associated with poor prognosis. Spearman correlation analysis revealed the correlation between GADD45G and VIM. Further experiments revealed that GADD45G modulates glioma cell invasion and migration, potentially through its effects on EMT-like phenotypic features.

**Conclusion:**

GADD45G expression is significantly associated with glioma outcomes and may serve as a promising biomarker for prognosis evaluation. Its involvement in regulating EMT-like phenotypic traits further highlights its therapeutic potential.

## Background

Gliomas constitute approximately 50% of primary central nervous system tumors. Despite advancements in surgical techniques and neuro-oncology, the prognosis for gliomas remains dismal. Glioblastoma multiforme (GBM), the most malignant variant of glioma, exhibits a 1-year survival rate of approximately 30% and a 5-year survival rate of less than 5% (1). Although surgical resection, combined with radiotherapy and chemotherapy, constitute common treatment modalities, the survival rates for certain glioma patients remain low. Therefore, it is imperative to investigate the oncogenic molecular mechanisms underlying gliomas and to develop effective targeted gene therapies to enhance patient outcomes.

The growth arrest and DNA damage-inducible gene (GADD45) family, consisting of GADD45A, GADD45B, and GADD45G, is involved in various fundamental processes such as DNA repair (2), genomic stability (3), epigenetic regulation (4), cell cycle arrest (5), apoptosis (6), tumorigenesis (7), and embryogenesis (6). Studies have indicated that GADD45 proteins regulate tumor progression under oncogenic stress (3, 8, 9). In esophageal cancer cell lines, GADD45G is silenced by promoter methylation, whereas GADD45A and GADD45B are not affected (10). The methylation status and protein expression of GADD45G are significantly associated with the survival of esophageal squamous cell carcinoma (ESCC) patients, while the expression of GADD45A and GADD45B is not (10). GADD45G expression can be induced by cellular stress and cytokine subsets (11–13). Research indicates that the expression of GADD45G is frequently reduced in anaplastic thyroid carcinoma (14), gastric cancer, colorectal cancer, pancreatic cancer (15), non-Hodgkin lymphoma, Hodgkin lymphoma, nasopharyngeal carcinoma, cervical cancer, esophageal cancer, and lung cancer (16). GADD45G may play a crucial role in tumorigenesis. Although dysregulated expression of GADD45G has been observed in several human tumors, its role in gliomas remains unclear. Therefore, investigating the role of GADD45G in glioma and its underlying mechanisms could uncover novel therapeutic targets, potentially enhancing the survival rate and clinical prognosis of glioma patients.

In this study, we conducted a comprehensive analysis of gliomas using bulk and single-cell RNA sequencing data, and further explored the potential role of GADD45G in glioma progression through *in vitro* experiments.

## Materials and methods

### Gene expression and survival analysis

The pan-cancer datasets TCGA, TARGET, and GTEx, which have been unified and standardized, were downloaded from the UCSC database (https://xenabrowser.net/). Expression data and survival information were subsequently obtained after appropriate filtering. In addition, data from the CGGA database (http://www.cgga.org.cn/) for glioma, as well as the glioma-related dataset (GSE108476) from the GEO database (https://www.ncbi.nlm.nih.gov/geo/), were integrated. We conducted a multivariate Cox regression analysis of GADD45G using TCGA GBM data to assess its potential as an independent prognostic indicator for glioblastoma. Based on this model, individual risk scores were calculated, and patients were subsequently stratified into high-risk and low-risk groups. Survival analyses of the CGGA, GSE108476, and TCGA datasets were performed using gliovis (https://gliovis.bioinfo.cnio.es/), employing Kaplan-Meier survival curves to evaluate the relationship between gene expression and overall survival (OS). The log-rank test was applied to assess the statistical significance of differences between survival curves.

### ScRNA-seq data processing and integration

The scRNA-seq data from the GEO database, including GSE103224, GSE138794, and GSE173278, were analyzed using the Seurat package in R 4.2.2. Cells with fewer than 200 total feature RNAs, considered low-quality, were excluded. After normalizing gene expression for each cell, batch correction was conducted using the Harmony package, and the batch effects were assessed based on clustering of different cell types (17). Dimensionality reduction was performed using principal component analysis (PCA), followed by uniform manifold approximation and projection (UMAP). Following the application of the FindNeighbors and FindClusters functions, 29 clusters were initially identified. Manual annotation was performed based on typical markers, and small clusters were subsequently merged. A total of 98,216 cells were included, with six major cell clusters identified as Malignant cell, Endothelial, Tumor-Associated Macrophages, Pericyte, Oligodendrocyte, and T cell for subsequent analysis. After extracting the manually annotated Malignant cell cluster, five major subclusters were identified: MES-like, AC-like, NPC-like, OPC-like, and unknown (18, 19). Marker gene sets for MES-like, AC-like, NPC-like, and OPC-like cells were obtained (20), and cell feature scoring was conducted using the AddModuleScore function. Although batch correction was applied using the Harmony algorithm and clustering of cell types showed satisfactory alignment, some residual variability may still exist. In addition, as the analysis was based on publicly available datasets, the sample representation may be somewhat modest, which could influence the generalizability of single-cell findings.

### Inference of CNV and downstream analysis in typical malignant cells

To differentiate malignant cells from normal cells, 400 cells from each cell type were isolated, and copy number variations (CNV) were inferred using the infercnv package. Genes with low expression (median expression < 0.1) were excluded. Genes were annotated based on their chromosomal locations, and CNV scores were computed using a moving average of 100 genes. Hierarchical clustering was conducted to distinguish non-malignant cells from malignant cells characterized by distinct chromosomal deletions or amplifications. A total of 1,000 cells from each typical malignant subpopulation were isolated to construct a single-cell reference matrix. We performed phylogenetic analysis of the four single-cell subtypes using the BuildClusterTree function to construct a phylogenetic tree. Cell-type proportions in GBM patients from the CGGA dataset were estimated using the default settings of CIBERSORTX (https://cibersortx.stanford.edu/). Subsequently, survival outcomes for different cell types were assessed using the survminer package. Gene Ontology (GO) enrichment analysis and Gene Set Variation Analysis (GSVA) were conducted to assess the functional heterogeneity of the four typical subpopulations. CNV data were obtained from the TCGA-GBM project via TCGAbiolinks, and GISTIC2 (https://broadinstitute.github.io/gistic2/) was employed for analysis. The GeoTcgaData and tinyarray packages were utilized to group the CNV data and conduct differential analysis. The processed CNV data were integrated with gene expression data, and CNV scores and expression values for target genes were extracted to construct a unified data frame for analysis. Finally, survival outcomes at varying CNV levels were assessed using the survminer package.

### Correlation analysis

Single-cell transcriptomic data were used to extract the expression levels of the target gene GADD45G, which were then subjected to Spearman correlation analysis. The ten genes most strongly correlated with GADD45G were identified. The expression patterns of these selected genes were visualized on a UMAP plot using the FeatureDimPlot function from the SCP package.

### Immune cell infiltration analysis

Based on the bulk RNA-seq expression profiles of tumor tissue samples in the TCGA-GBM dataset, the relative abundance of immune cells in the tumor microenvironment was estimated using the single-sample gene set enrichment analysis (ssGSEA) method. The signature gene sets for various immune cell types were constructed from their representative marker genes. Through enrichment analysis, enrichment scores of each immune cell type were obtained for every sample. Subsequently, Spearman correlation analysis was performed to assess the relationship between GADD45G expression levels and the degree of immune cell infiltration.

### Drug analysis

Drugs capable of regulating the transcriptional expression levels of GADD45G were identified from the CTD database (https://ctdbase.org/). Drug sensitivity analysis for GADD45G was conducted using an online database (https://guolab.wchscu.cn/GSCA/#/).

### Cell culture and transfection

The SKMG1 cell line was maintained by the State Key Laboratory of Oncology in South China, while the A172 cell line was provided by Dr. Shing-shun Tony To, from the Department of Health Technology and Informatics, The Hong Kong Polytechnic University. All cell lines were authenticated by short tandem repeat (STR) profiling within the last six months. The cells were cultured in Dulbecco’s Modified Eagle Medium (DMEM; Carlsbad, USA) supplemented with 10% fetal bovine serum (FBS; Gemini Bio-Products, West Sacramento, CA, USA), 100 U/mL penicillin-streptomycin (HyClone, Logan, UT, USA), and 2 mM glutamine (HyClone). Cells were maintained at 37 °C in 5% CO_2_. The plasmid pCDNA3.1-CMV-GADD45G-3xFLAG-hGHpolya-EF1a-EGFP, constructed by Obio Technology (Shanghai, China), was used to overexpress the GADD45G gene (GenBank ID: NM_006705.4, 480 bp). Cells were seeded at 5 × 10^5 per well in 6-well plates and transfected at 60% confluency. The transfection mixture (2.5 µg plasmid and 5 µL Lipofectamine 3000) was incubated with cells for 48 hours. For the control group, cells were transfected with the empty vector H23990 pCDNA3.1-CMV-MCS-3xFLAG-hGHpolya-EF1a-EGFP (Obio Technology, Shanghai, China) under identical conditions to the experimental group.

### Reverse transcription quantitative PCR

Total RNA was extracted using the RNA extraction kit (ESScience) following the manufacturer’s instructions. The RNA concentration was measured, and complementary DNA (cDNA) was synthesized via reverse transcription. This was followed by amplification and quantitative analysis of the cDNA using real-time PCR (qPCR) technology. The primers were synthesized by Sangon Biotech (Shanghai, China). The primer sequences were as follows: GADD45G: 5′-CAGATCCATTTTACGCTGATCCA-3′ (forward) and 5′-TCCTCGCAAAACAGGCTGAG-3′ (reverse). Glyceraldehyde 3-phosphate dehydrogenase (GAPDH) served as the internal control.

### Western blot analysis

Proteins were separated by SDS-PAGE and transferred to PVDF membranes. The membranes were blocked with 5% non-fat milk and incubated overnight at 4°C with the primary antibody (GADD45G, 1:1000, Santa Cruz Biotechnology). Afterward, the membranes were incubated for 2 hours with a secondary antibody (1:5000). Protein levels were detected using a chemiluminescence and fluorescence imaging system.

### Scratch assay

Cells were cultured in dishes until they reached 80%-90% confluency. A straight-line scratch was created in the monolayer using a cell scraper. Photographs of the scratch area were taken at 0, 24, and 48 hours to document the healing process.

### Transwell migration assay

A cell suspension in serum-free medium was added to the upper chamber of the Transwell insert at a density of 5×10^4^ cells per well. The lower chamber was filled with medium supplemented with 10% fetal bovine serum (FBS) as a chemoattractant. The Transwell chambers were incubated at 37°C with 5% CO_2_ for 24 hours. After incubation, non-migrated cells on the upper surface were gently removed with a cotton swab. Migrated cells on the lower surface were fixed with 4% paraformaldehyde, stained, and then observed and counted under a microscope.

### Transwell invasion assay

Cell suspensions (5×10^4^ cells per well) were added to the upper chamber of Matrigel-coated Transwell inserts in serum-free medium. The lower chamber was filled with medium containing 10% fetal bovine serum (FBS) to serve as a chemoattractant. The Transwell inserts were incubated at 37°C in a 5% CO_2_ incubator for 24 hours. After incubation, the upper surface of the inserts was gently wiped with a cotton swab. Cells that had invaded the lower surface were fixed with 4% paraformaldehyde, stained, and subsequently observed and counted under a microscope.

### CCK-8 assay

A172 and SKMG1 cells were seeded at 2,000 cells/well in 96-well plates and cultured for 24 – 96 hours. Then, 10 µL of CCK - 8 reagent (Dojindo) was added per well and mixed. The plates were incubated for 1 hour. Absorbance was measured at 450 nm, followed by statistical analysis.

### Cell apoptosis assay

The apoptosis assay was performed using the Annexin V-FITC Apoptosis Detection Kit (Beyotime) following the manufacturer’s instructions. Cells were first collected and washed twice with PBS. The cells were then resuspended in 1X Annexin V binding buffer, and 5 µL of Annexin V-FITC and 5 µL of PI were added to each sample. The samples were gently mixed and incubated in the dark at room temperature for 15 minutes. After incubation, the apoptosis rate was measured using a flow cytometer.

### Statistics and analysis

Statistical analyses and graphical representations were performed using R (v4.3.1), Python (v3.10.9), and GraphPad Prism (v8.3). Kaplan-Meier survival analysis was conducted using the log-rank test, and Pearson’s correlation coefficient was utilized to assess linear relationships. Comparisons between two groups were performed using Student’s t test, while comparisons among three or more groups were performed using one-way analysis of variance (ANOVA). P-values are shown within the plots to indicate statistical significance (*P < 0.05, **P < 0.01, ***P < 0.001, ****P < 0.0001, ns: not significant).

## Result

### Differential expression of GADD45G in glioma

**Figure 1:**
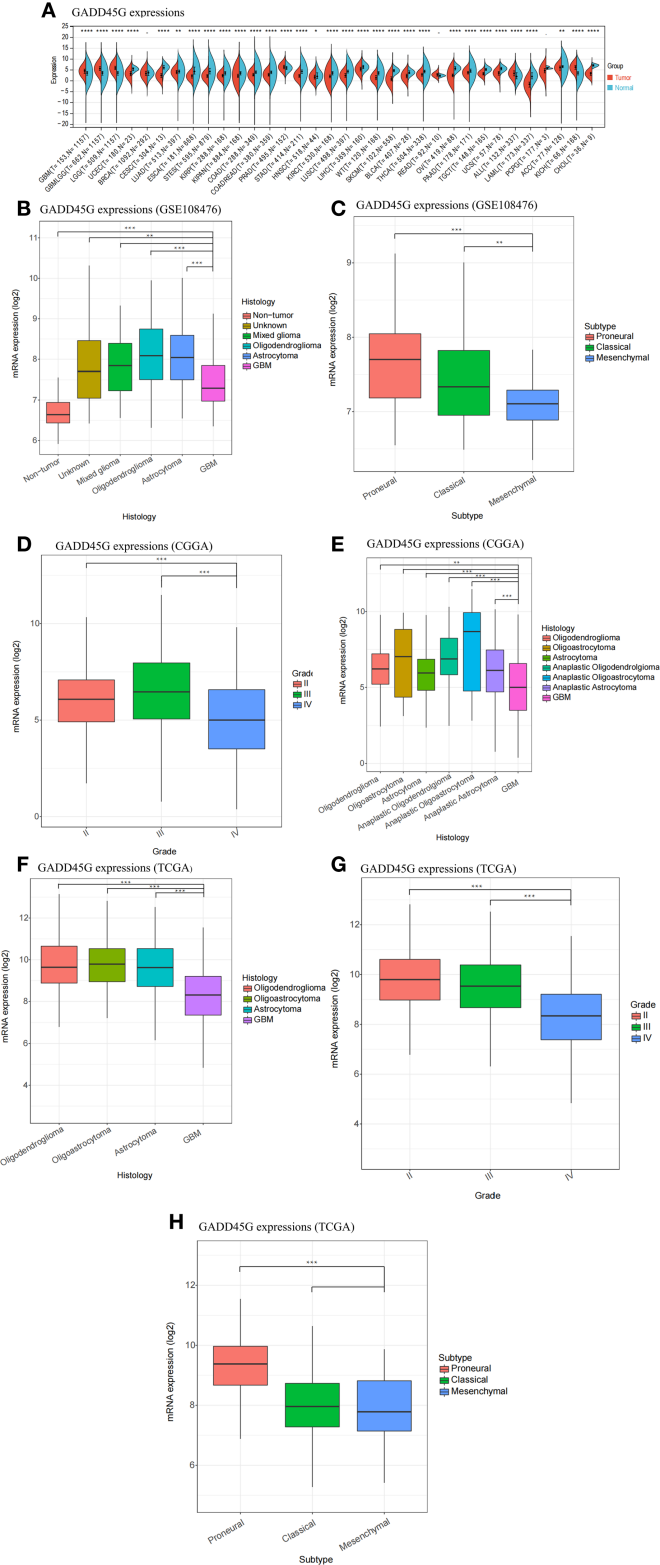
Differential expression of GADD45G in gliomas. **(A)** Expression of GADD45G across multiple cancer types (pan-cancer). **(B–H)** Transcriptional expression levels of GADD45G in glioma samples based on multiple datasets, including GSE108476 **(B, C)**, CGGA **(D)**, and TCGA **(F–H)**. The full names corresponding to each cancer code are provided in the **Supplementary Table 1** for reference. P-values are shown within the plots to indicate statistical significance (*P < 0.05, **P < 0.01, ***P < 0.001, ****P < 0.0001, ns, not significant).

We analyzed the expression of GADD45G across various tumors and corresponding normal tissues using pan-cancer datasets from TCGA, TARGET, and GTEx (**Figure 1A**). To facilitate interpretation, we listed the full names corresponding to each cancer code shown in **Figure 1A** in **Supplementary Table 1**. **Supplementary Figure S1** further illustrates the hazard ratios of GADD45G across different cancer types. The results showed that GADD45G expression was significantly downregulated in most tumors compared to normal tissues (**Figure 1A**). Notably, gliomas exhibited an opposite trend: GADD45G expression was higher in glioma tissues than in normal brain tissues (**Figure 1A**). Furthermore, we observed that GADD45G expression in low-grade gliomas (LGG) was higher than that in glioblastoma (GBM). Similar results were validated in three independent datasets, including GEO (**Figures 1B, C**), CGGA (**Figures 1D, E**), and TCGA (**Figures 1F–H**). In particular, comparisons across different histological types and WHO grades consistently showed that GADD45G expression was lower in GBM compared to lower-grade gliomas.

### GADD45G as a prognostic marker for favorable outcome in glioma

**Figure 2:**
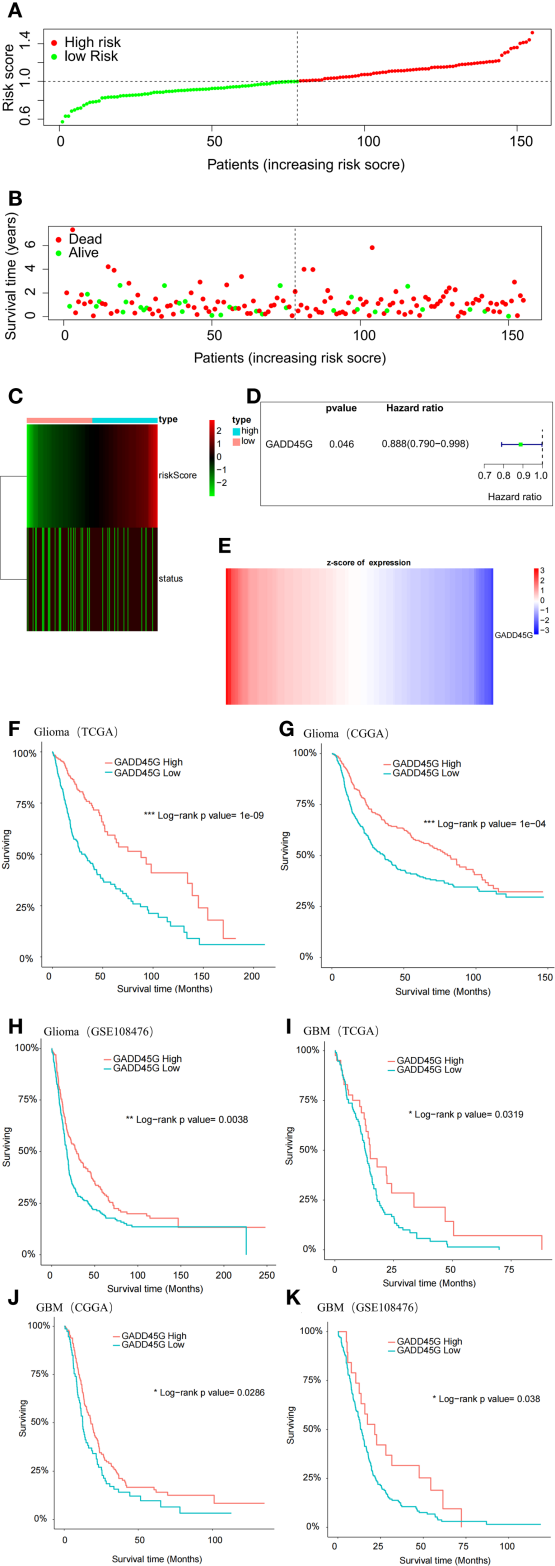
Prognostic significance of GADD45G in gliomas. **(A)** Multivariate Cox analysis based on TCGA-GBM data. **(B, C)** Survival status analysis based on risk stratification. **(D)** Hazard ratio analysis of GADD45G. **(E)** Expression analysis of GADD45G across risk groups. **(F–K)** Kaplan–Meier survival curves of overall survival (OS) for patients with different levels of GADD45G transcriptional expression based on TCGA, CGGA, and GSE108476 datasets.

To assess the prognostic significance of GADD45G in glioma, we conducted a multivariate Cox regression analysis on TCGA-GBM data, categorizing patients into high-risk and low-risk groups based on their computed risk scores (**Figure 2A**). The analysis revealed that the low-risk group had a higher proportion of surviving patients, while the high-risk group exhibited an increased proportion of deceased patients (**Figures 2B, C**). Cox regression analysis revealed that patients with high GADD45G expression exhibited an 11.2% reduction in mortality risk compared to those with low expression, suggesting a potential protective role for GADD45G (**Figure 2D**). When patients were stratified by ascending risk scores, GADD45G expression levels exhibited a progressive decline (**Figure 2E**). To further validate our findings, Kaplan-Meier survival analysis was conducted. The results demonstrated that, across all datasets, higher GADD45G expression was significantly correlated with improved overall survival (OS) in both glioma and glioblastoma, suggesting that GADD45G may serve as a favorable prognostic biomarker for glioma patients (**Figures 2F–K**). The study of the correlation between GADD45G expression and the infiltration levels of various immune cells suggests that it may influence the progression of glioma by affecting myeloid-derived suppressor cells, activated CD4 T cells, and CD56bright natural killer cells (**Supplementary Figure S2**).

### Analysis of tumor microenvironment through single-cell data

**Figure 3:**
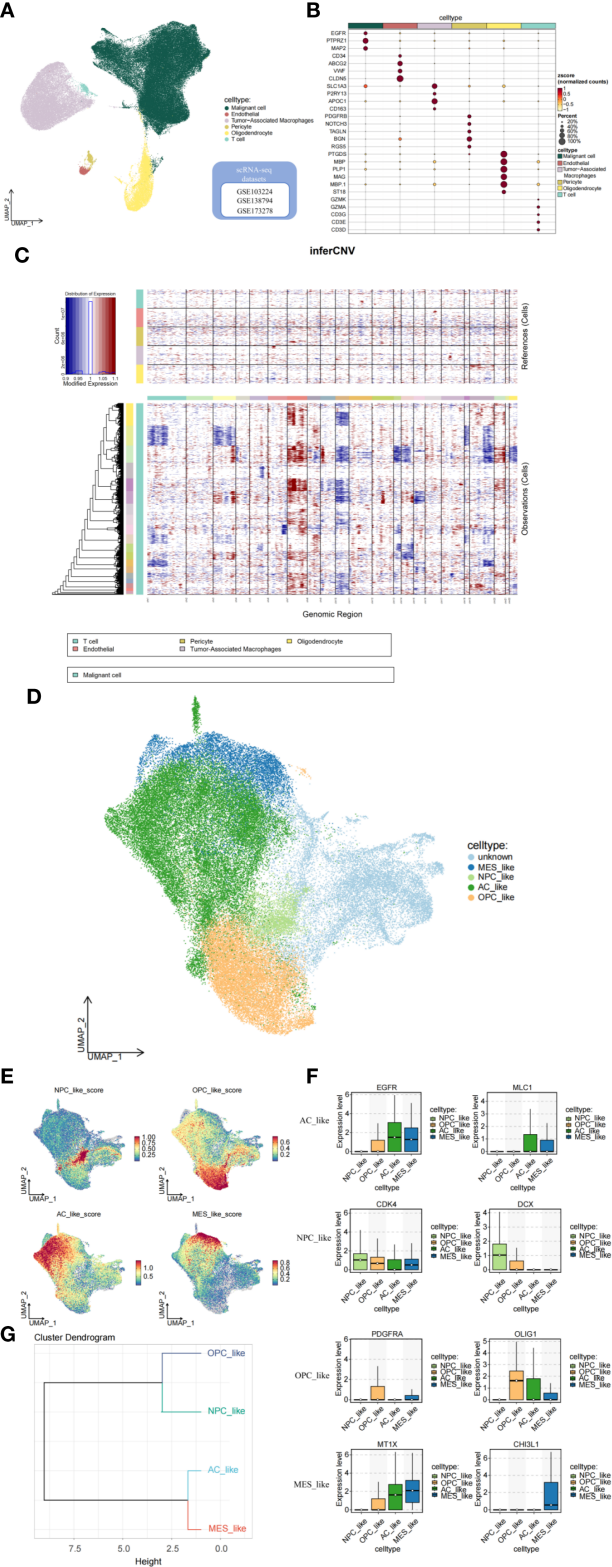
Analysis of the tumor microenvironment based on single-cell data. **(A, B)** Cell clusters and their marker genes. **(C)** CNV analysis indicating tumor cell characteristics. **(D, E)** Subgrouping and annotation of malignant GBM cells. **(F)** Marker genes of GBM malignant cell subpopulations. **(G)** Dendrogram of cell subcluster relationships.

Conventional bulk transcriptome analysis cannot fully capture the characteristics of different tumor cell subtypes. Therefore, we further investigate the relationship between GADD45G and glioblastoma (GBM) by utilizing single-cell data. We integrated GBM scRNA-seq data collected from three independent datasets and analyzed a total of 98,216 cells. Six important cell clusters were color-coded and labeled as shown in the figure (**Figure 3A**). The bubble plot displays the marker genes for each major cell type (**Figure 3B**). Copy number variation (CNV) analysis indicates that, compared to normal cells, tumor cells exhibit chromosome 7 amplification and chromosome 10 deletion, which is consistent with previous studies (21) (**Figure 3C**). The UMAP plot reveals five distinct cellular subpopulations within malignant GBM cells (**Figure 3D**). To evaluate the heterogeneity among GBM subpopulations, we calculated the geometric mean of gene feature expression. This approach provides a quantitative measure of variability within distinct cellular subtypes, allowing for a more comprehensive characterization of intratumoral diversity (**Figure 3E**). According to Neftel’s classification, GBM cells primarily comprise four distinct cell types: neural progenitor-like (NPC-like), oligodendrocyte progenitor-like (OPC-like), astrocyte-like (AC-like), and mesenchymal-like (MES-like) cells (18). The box plot illustrates the transcriptional expression of marker genes across four malignant GBM cell subpopulations (**Figure 3F**). From a developmental perspective, AC-like and MES-like cells exhibit greater similarity to each other, while OPC-like and NPC-like cells are more closely related (**Figure 3G**).

### Characterization of malignant GBM subpopulations

**Figure 4:**
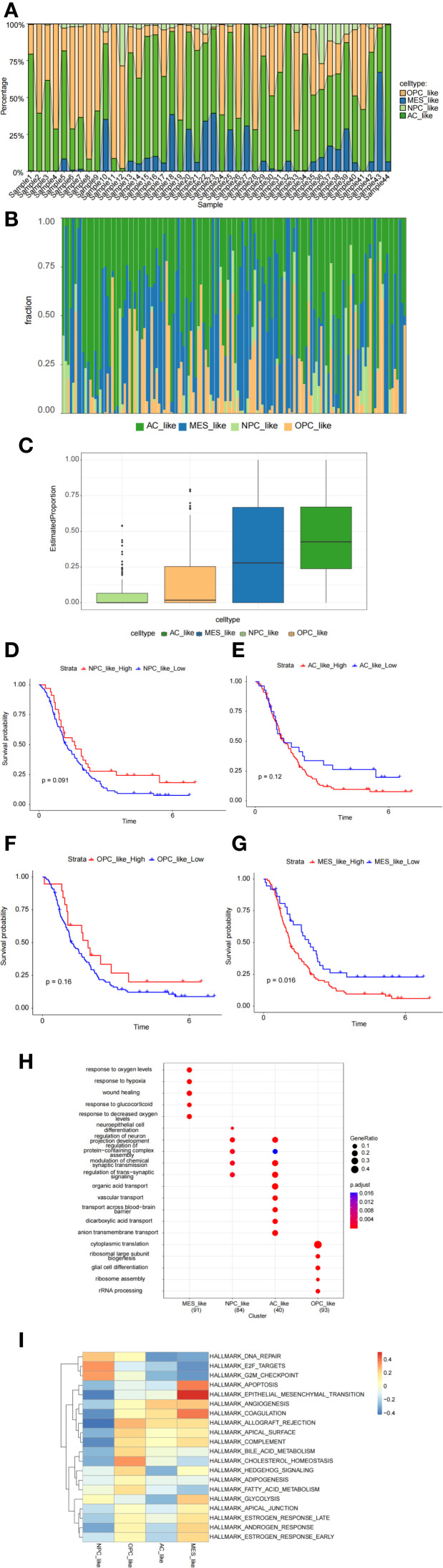
Characterization of malignant cell subpopulations. **(A)** Proportion analysis of malignant cell subpopulations based on single-cell data. **(B, C)** Proportion analysis of malignant cell subpopulations based on CGGA data. **(D–G)** Kaplan–Meier survival analysis exploring the prognostic significance of MES-like cells. **(H, I)** Gene Ontology (GO) enrichment analysis and gene set variation analysis (GSVA) revealing functional heterogeneity among cell subpopulations.

The proportions of cell subtypes vary significantly across different samples, reflecting the heterogeneity of GBM (**Figure 4A**). The CGGA dataset further supports our findings, reinforcing the robustness of our results (**Figures 4B, C**). Overall, the molecular subtypes identified at the single-cell level indicate that GBM patients exhibit a high degree of intratumoral subtype heterogeneity. The Kaplan-Meier survival analysis indicates that higher expression of MES-like cells is associated with poorer survival outcomes (**Figures 4D–G**). Gene Ontology (GO) enrichment analysis and Gene Set Variation Analysis (GSVA) were used to determine the functional heterogeneity of the four typical subgroups. The results indicated that MES-like cells were significantly enriched in biological processes associated with hypoxia and wound healing. An improved capacity to adapt to hypoxic conditions promotes tumor progression and invasion. The enrichment of wound healing-related functions suggests that MES-like cells may possess enhanced proliferative and migratory potential. Additionally, the enrichment of glucocorticoid response-related functions may contribute to immune evasion mechanisms (**Figure 4H**). Furthermore, MES-like cells were highly enriched in the HALLMARK_EPITHELIAL_MESENCHYMAL_TRANSITION (EMT) pathway, which is critically linked to cellular invasion, migration, and malignant progression. These findings indicate that GBM patients exhibiting mesenchymal characteristics tend to have worse survival outcomes (**Figure 4I**).

### Expression levels of GADD45G and its correlated genes in malignant GBM cells

**Figure 5:**
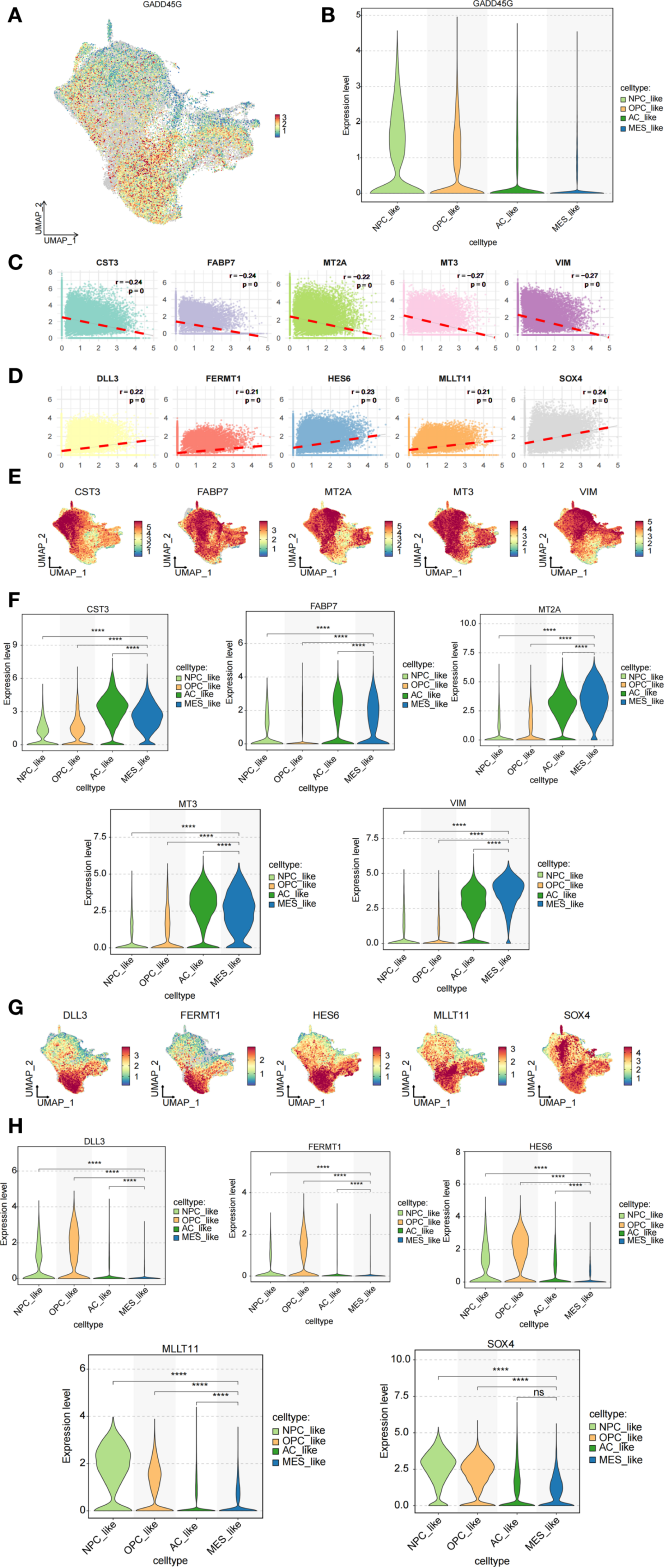
Analysis of GADD45G and its related genes in cell subpopulations. **(A, B)** Expression analysis of GADD45G across different cell subpopulations. **(C, D)** Spearman correlation analysis identifying highly correlated genes. **(E–H)** Expression analysis of the correlated genes across cell subpopulations.

To examine the expression pattern of GADD45G across distinct cellular subpopulations in GBM, we employed single-cell RNA sequencing (scRNA-seq) data to map its distribution among NPC-like, OPC-like, AC-like, and MES-like cell populations (**Figures 5A, B**). The analysis revealed that GADD45G exhibited the lowest expression levels in MES-like cells. Notably, this finding aligns with our results from other databases, which consistently indicate a downregulation of GADD45G in malignant cells. In order to identify and develop small molecule drugs capable of inducing GADD45G expression in cancer cells, we examined the correlation between GADD45G expression and various cancer-related drugs using the CTD database (**Supplementary Figure S3**). To elucidate the functional role of GADD45G in GBM, we conducted Spearman correlation analysis to identify genes strongly associated with GADD45G, thereby uncovering potential regulatory networks. The figure illustrates the top 10 genes most strongly correlated with GADD45G (**Figures 5C, D**). Among these genes, MT3 and VIM demonstrated the strongest inverse correlation with GADD45G (r = -0.27), indicating that their expression may be downregulated by GADD45G. Through the analysis of single-cell transcriptomic data, we discovered that VIM was markedly upregulated in MES-like cells relative to other cell populations (**Figures 5E–H**).

### The role of GADD45G in cell invasion and migration in glioma through regulation of EMT-like phenotypes

**Figure 6:**
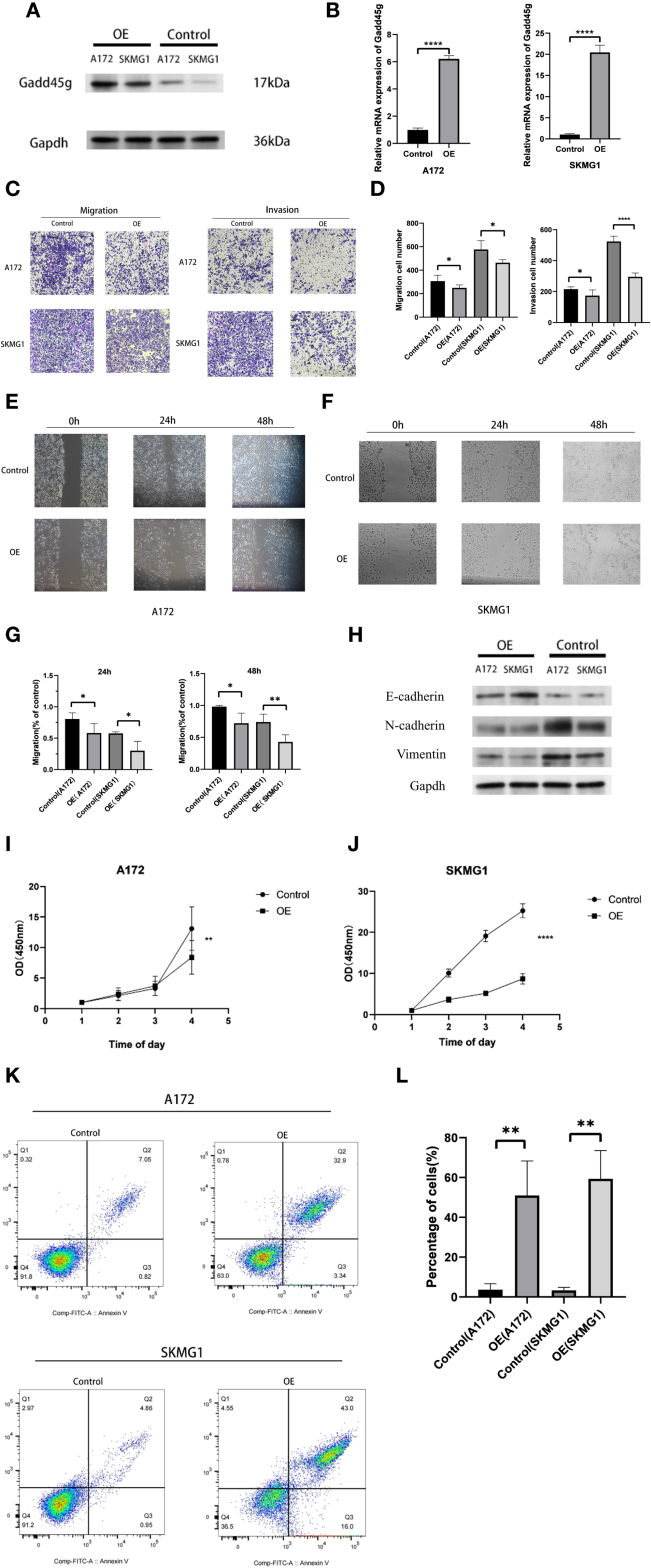
Functional effects of GADD45G on glioma cells. **(A)** Western blot analysis of GADD45G expression in A172 and SKMG1 cells after plasmid transfection. **(B)** RT-qPCR analysis of GADD45G expression in A172 and SKMG1 cells after plasmid transfection. **(C, D)** Transwell migration and invasion assays and their quantification in A172 and SKMG1 cells. **(E–G)** Wound healing assays showing the migration ability of A172 and SKMG1 cells at 0, 24, and 48 hours and their quantification. **(H)** Western blot analysis of EMT-like phenotypic markers. **(I, J)**. CCK - 8 assay evaluating changes in cell proliferation of A172 and SKMG1 cells. **(K, L)**. Flow cytometry analysis of apoptosis in A172 and SKMG1 cells. *P < 0.05, **P < 0.01, ***P < 0.001, ****P < 0.0001.

In the present study, we demonstrated that MES-like cells are significantly enriched in the EMT-related gene sets, and Spearman correlation analysis revealed a strong negative correlation between GADD45G and VIM. Although gliomas are not epithelial in origin and do not undergo classical epithelial-to-mesenchymal transition (EMT), they can exhibit an EMT-like transcriptional program characterized by loss of polarity, enhanced motility, and expression of mesenchymal markers (22–24). Based on this concept, we hypothesized that GADD45G might regulate glioma progression by modulating EMT-like features. To test this, we performed plasmid transfection experiments in glioma cell lines A172 and SKMG1. Western blotting and RT-qPCR confirmed successful overexpression of GADD45G (**Figures 6A, B**). Following GADD45G overexpression, both cell lines showed significantly reduced invasion and migration abilities in Transwell and wound healing assays (**Figures 6C–G**). Epithelial-mesenchymal transition (EMT) plays a key role in tumor metastasis, acting as a critical driver for the invasion and migration of tumor cells (25, 26). To further investigate whether GADD45G affects EMT-like marker expression, we analyzed E-cadherin, N-cadherin, and vimentin levels by Western blotting (**Figure 6H**). Overexpression of GADD45G increased E-cadherin expression while suppressing mesenchymal markers N-cadherin and vimentin. These results suggest that GADD45G attenuates glioma cell invasiveness in part by regulating EMT-like phenotypic traits. In conclusion, although gliomas do not undergo canonical EMT, our data support that GADD45G plays a role in modulating EMT-like processes that contribute to tumor cell migration and invasion. To further investigate the biological effects of GADD45G overexpression in glioma cells, we performed CCK - 8 proliferation assays and Annexin V-FITC/PI double-staining flow cytometry to assess its impact on cell proliferation and apoptosis. As shown in **Figures 6I, J**, overexpression of GADD45G significantly inhibited the proliferation of A172 and SKMG1 glioma cells compared to the control group. Meanwhile, flow cytometry results demonstrated that GADD45G overexpression markedly increased the apoptotic rate in both cell lines (**Figures 6K, L**), suggesting that GADD45G not only suppresses glioma cell growth but also induces apoptosis. These findings indicate that GADD45G exerts tumor-suppressive functions in glioma by simultaneously inhibiting proliferation and promoting apoptosis.

## Discussion

Gliomas, particularly Glioblastoma multiforme (GBM), are characterized by high invasiveness, rapid growth, and the ability to spread to other brain regions, which complicates complete surgical resection. An urgent need exists for the development of novel early diagnostic and prognostic targets to overcome the challenges associated with glioma treatment. A significant body of research has focused on identifying potential biomarkers that could open new therapeutic avenues for gliomas (27–30). To the best of our knowledge, this is the first study to elucidate the role of GADD45G in gliomas.

Notably, during the development of various cancer types, GADD45G is underexpressed and is regarded as a functional tumor suppressor. Comprehensive analysis of multiple databases has revealed that GADD45G is highly expressed in glioma patients, suggesting its potential role as an oncogene. However, Cox regression analysis indicates that GADD45G possess a protective function. Survival curves further demonstrate that elevated levels of GADD45G are correlated with improved overall survival in patients. To explore this further, we modulated GADD45G expression using plasmid overexpression, thereby confirming its tumor-suppressive role in glioma. Notably, GADD45G expression is markedly reduced in glioblastoma relative to lower-grade gliomas. GADD45G expression can be induced by cellular stress and certain subsets of cytokines (11–13). Deficiencies in the GADD45 pathway have been implicated in the initiation and progression of malignant tumors (31, 32). We hypothesize that in low-grade gliomas, GADD45G upregulation may inhibit tumor progression, whereas, in malignant tumors, the suppression of GADD45G expression by tumor cells could contribute to tumor deterioration. To test this hypothesis, we conducted *in vitro* experiments where we overexpressed GADD45G in glioma cells. As anticipated, GADD45G overexpression led to a significant inhibition of glioma cell migration and proliferation.

Prior to the advent of single-cell RNA sequencing (scRNA-seq), traditional transcriptomic approaches were the primary methods used for identifying prognostic biomarkers and anti-cancer targets. However, bulk RNA sequencing, which cannot capture the expression profiles of individual cells, is limited in its ability to detect the tumor microenvironment at the cellular level. Therefore, we utilized scRNA-seq data from GBM samples to investigate the heterogeneity within GBM tumor subgroups. In this study, we systematically identified four highly heterogeneous GBM cell subgroups through a series of stringent protocols. In contrast, MES-like cells were predominantly characterized by epithelial-mesenchymal transition (EMT), hypoxic conditions, and wound healing pathways. Survival analysis revealed a significant association between the mesenchymal (MES) subtype and poor patient prognosis, suggesting that the MES subtype may represent the most aggressive form of glioblastoma. As demonstrated in the study by Yang et al., the MES subtype corresponds to the terminal stage of GBM cellular evolution (33). To further elucidate the role of GADD45G, we examined its expression across distinct GBM cellular subpopulations. Consistent with trends observed in bulk transcriptomic data, GADD45G expression was notably suppressed in malignant cell populations, further supporting our prior hypothesis. To explore the functional role of GADD45G in GBM, we conducted Spearman correlation analysis and examined the transcriptional expression levels of related genes across four distinct cell subpopulations. Notably, we observed that VIM expression was significantly elevated in the MES cell population compared to other subpopulations. In the context of oncology, Vimentin is widely recognized as a mesenchymal marker and is strongly upregulated during epithelial-mesenchymal transition (EMT), a process critical to tumor invasion and metastasis. We observed a significant inverse correlation between GADD45G and VIM expression.

Although gliomas are not epithelial in origin, the concept of an epithelial-to-mesenchymal transition-like (EMT-like) process has been increasingly discussed in the context of glioma biology, particularly to describe transcriptional programs associated with enhanced cell plasticity and invasion. EMT-like programs refer not to a full transdifferentiation between epithelial and mesenchymal cell states, but rather to a transcriptional and phenotypic shift that promotes tumor cell plasticity, invasiveness, and resistance to treatment. This concept is supported by transcriptomic analyses showing that glioma cells, including primary GBM and established cell lines, express a mesenchymal signature comparable to TGF-β-induced EMT in epithelial tumors (34). EMT-like markers and pathways, including Snail, ZEB1/2, Twist, and Wnt/TGF-β signaling, have been widely detected in glioblastoma and are implicated in tumor progression (35–43). Therefore, the observed relationship between GADD45G and EMT markers in our study is not indicative of classical EMT, but rather reflects a regulatory interaction within this EMT-like phenotypic modulation that is biologically relevant in glioma. Based on this conceptual framework, we next sought to investigate whether GADD45G influences EMT-like features in glioma cells through experimental validation. We subsequently conducted cell-based experiments to determine whether GADD45G modulates EMT and thereby influences the invasive and metastatic behavior of glioma cells. Overexpression of GADD45G reduced cell invasion and migration, and the protein levels of EMT markers were also downregulated. Overall, these results strongly support the role of GADD45G in inhibiting tumor metastasis. In fact, GADD45G has been studied in various cancers. Wei Guo et al. reported that reduced expression of GADD45G is associated with tumor progression and poor prognosis in esophageal squamous cell carcinoma (10). Xinbao Zhang et al. demonstrated that GADD45G initiates the differentiation of embryonic stem cells and inhibits carcinogenesis in breast cancer cells (44). Other studies have also suggested that GADD45G, a novel vitamin D-regulated gene, exerts anti-proliferative effects in prostate cancer cells (45). In conclusion, GADD45G may serve as a promising biomarker for cancer diagnosis and therapy.

p53 is a classical tumor suppressor that plays a pivotal role in cellular stress responses, DNA repair, apoptosis, and cell cycle regulation. It can inhibit tumor growth by inducing apoptosis or causing cell cycle arrest (46). PTEN, a phosphatase, primarily exerts its tumor-suppressive effect through negative regulation of the PI3K/Akt signaling pathway. By dephosphorylating PIP3, PTEN reduces Akt activation, thereby inhibiting cell proliferation and promoting apoptosis (47). Similarly, members of the GADD45 family act as stress response genes that can be activated by various intracellular and extracellular stimuli, such as DNA damage and oxidative stress, to initiate downstream signaling pathways involved in tumor suppression (12, 48, 49). Our findings suggest that GADD45G may exert its anti-tumor effects through a dual mechanism of EMT inhibition and apoptosis induction, functionally resembling classical tumor suppressors like p53 and PTEN. Given its dual role in regulating both tumor cell invasiveness and survival, GADD45G holds promise as a potential therapeutic target and prognostic biomarker in various malignancies. Further investigation into its molecular mechanisms is warranted.

The Gadd45 family has been widely studied in the context of various cancers, such as pancreatic, hepatocellular, lung, cervical, and gastrointestinal cancers, as well as different types of lymphomas. These studies highlight the dysfunctional roles and regulatory mechanisms of the Gadd45 family in tumorigenesis. Consequently, the Gadd45 family has been identified as a promising target for cancer therapies (50). Emerging functional evidence suggests that GADD45 proteins act as tumor suppressors in response to various stimuli, linking multiple cellular signaling modules. Inducing the expression of GADD45 is a necessary step in mediating the anticancer activity of various chemotherapy drugs, while the loss of GADD45 may eliminate its function in cancer cells. NAC1-mediated downregulation of GADD45G has been shown to contribute to paclitaxel resistance in ovarian cancer cells (51). In a breast cancer cell line resistant to farnesyltransferase inhibitors (FTI), (-)-Xanthatin was found to induce GADD45G, which subsequently activated p38 and JNK pathways, leading to decreased cell proliferation and caspase-independent apoptosis. Research has shown that structurally varied NSAIDs can trigger apoptosis in ovarian, prostate, renal, breast, and stomach cancer cell lines by activating melanoma differentiation-associated gene-7/Interleukin-24 (mda-7/IL-24) (52–55). The induction and activation of mda-7/IL-24 by NSAIDs result in the upregulation of GADD45A and GADD45G, which are crucial for the execution of cancer cell death through JNK activation (52).

Our study uncovers the regulatory mechanism of tumor development mediated by GADD45G in glioma, offering a promising avenue for future research. Future research focused on understanding how GADD45 pathways are regulated by the tumor microenvironment and cancer stem cells will offer new opportunities to target and manipulate GADD45 function. Moreover, we explored the correlation between GADD45G and various cancer drugs through the CTD database. Many of these drugs were found to upregulate the expression of GADD45G, which is clearly a research avenue worth further exploration.

Although this study integrates multiple bioinformatics analyses and *in vitro* experimental validations, we acknowledge that these findings represent only the preliminary phase of a broader investigation. A major limitation is the lack of validation using animal models or clinical tissue samples, which is essential for supporting the translational potential of GADD45G as a biomarker or therapeutic target in glioma. While our cellular models revealed correlations between GADD45G expression and tumor progression as well as an EMT-like phenotype, further validation in animal models and patient-derived tissues is necessary. We plan to establish a tissue microarray (TMA) resource based on clinical glioma samples and conduct qPCR or protein-level analyses to assess associations between GADD45G expression and tumor grade, subtype, and prognosis, providing stronger evidence for its clinical utility. In parallel, *in vivo* models such as orthotopic xenografts will be used to investigate the role of GADD45G in tumor growth, metastasis, and therapeutic response. Additionally, our single-cell RNA sequencing (scRNA-seq) analysis, based on three public datasets with limited sample sizes, may be affected by residual technical or biological variability despite batch effect correction, and may not fully capture GBM’s intratumoral heterogeneity. We have addressed this limitation in the manuscript and plan to expand our scRNA-seq analyses to include more publicly available datasets with spatial information and treatment-specific contexts, as well as generate our own high-resolution scRNA-seq data from freshly collected clinical glioma samples, thereby enabling more controlled analyses of GADD45G expression across distinct cellular and microenvironmental contexts. Lastly, the use of public transcriptomic and clinical datasets may introduce confounding factors such as inconsistencies in sample processing, differences in sequencing platforms, and incomplete clinical annotations, which should be carefully addressed in future prospective studies. In summary, although our *in vitro* experiments provide preliminary insights into the regulatory role of GADD45G in glioma cell migration, invasion, and EMT-like transitions, further systematic mechanistic studies and validations using clinical specimens and animal models are required to fully establish its prognostic and therapeutic potential.

## Data Availability

The original contributions presented in the study are included in the article/**Supplementary Material**. Further inquiries can be directed to the corresponding author.
