# Supplementary figures and images for: GADD45G as a novel prognostic biomarker and therapeutic target in glioma: integrative analysis of bulk and single-cell RNA sequencing
Source: Front Oncol. 2025 Sep 11;15:1608710. doi: 10.3389/fonc.2025.1608710 (PMC12460128; doi:10.3389/fonc.2025.1608710)

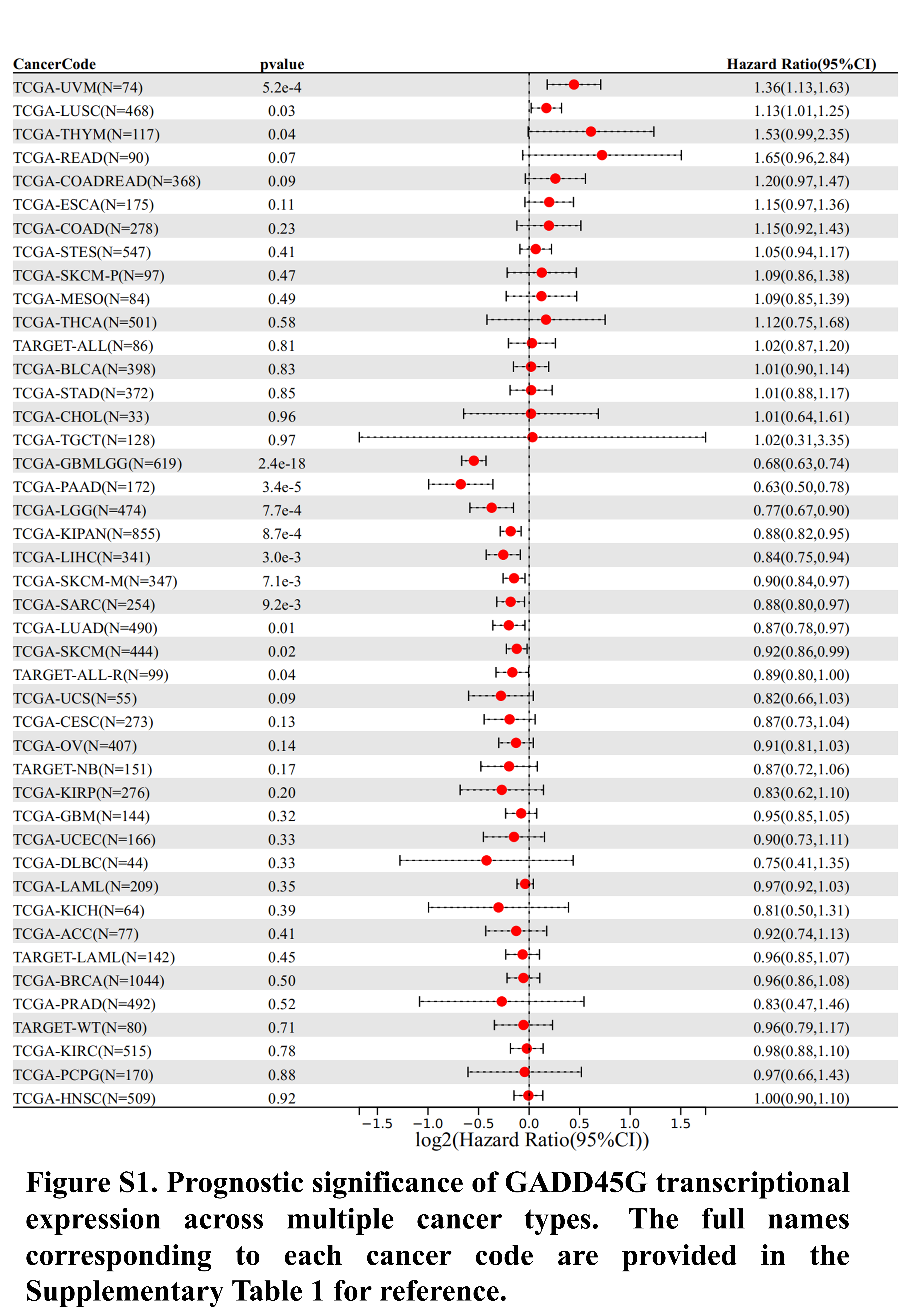

Supplement: Supplementary file 1 [file Image1.tif]

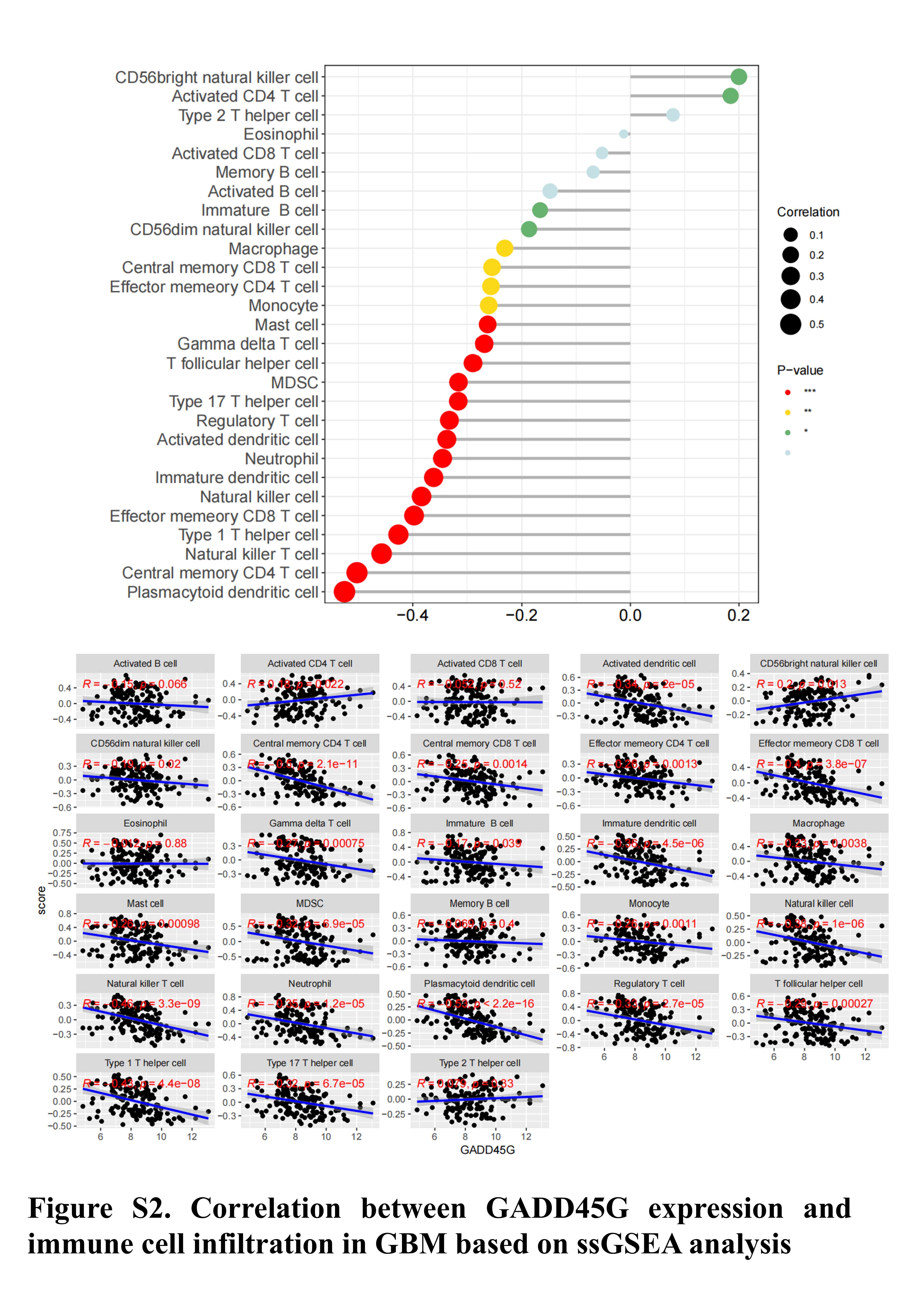

Supplement: Supplementary file 2 [file Image2.tif]

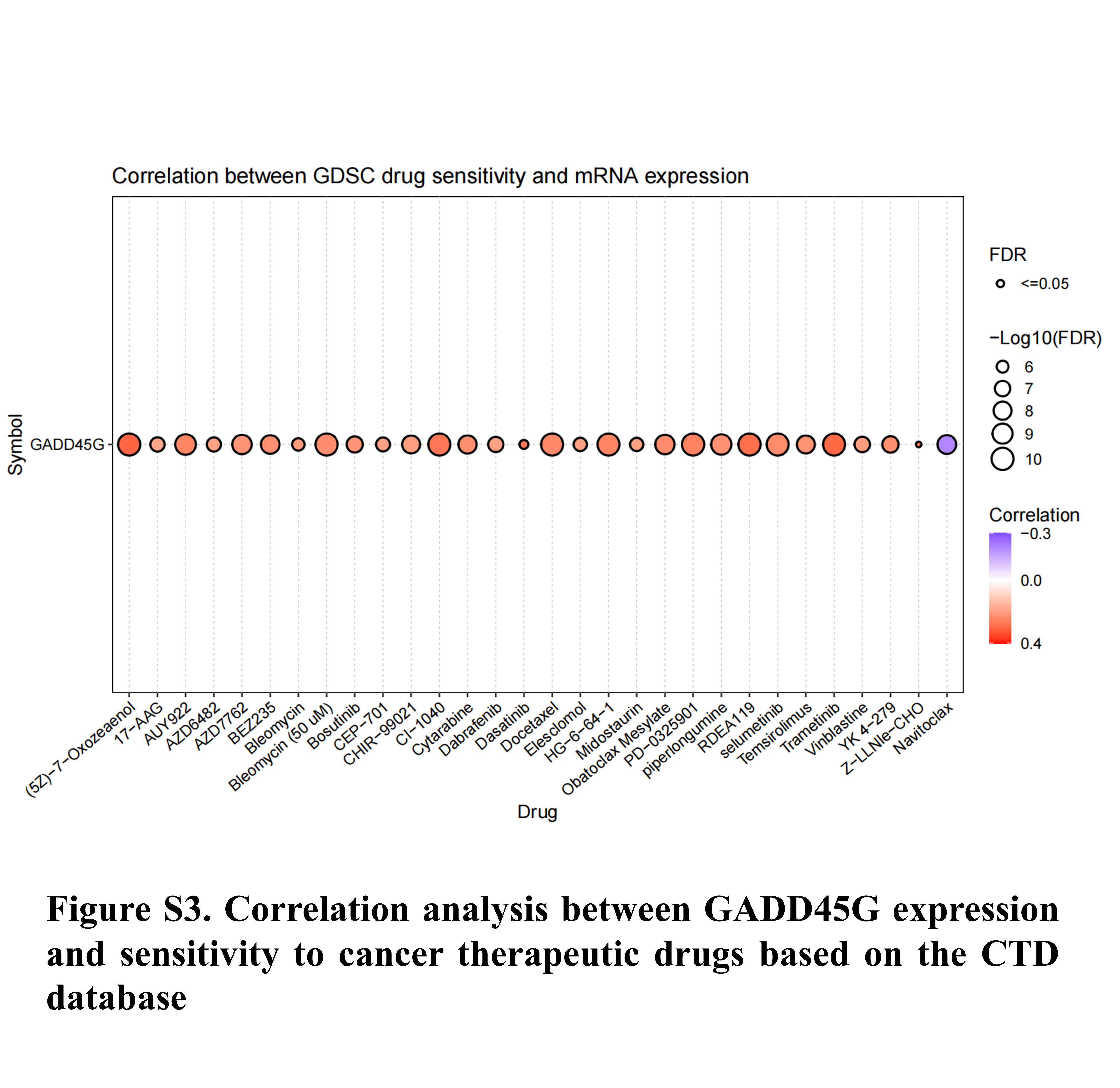

Supplement: Supplementary file 3 [file Image3.tif]
